# Analysis of volatile compounds production kinetics: A study of the impact of nitrogen addition and temperature during alcoholic fermentation

**DOI:** 10.3389/fmicb.2023.1124970

**Published:** 2023-03-07

**Authors:** Joséphine Godillot, Clara Baconin, Isabelle Sanchez, Meili Baragatti, Marc Perez, Yannick Sire, Evelyne Aguera, Jean-Marie Sablayrolles, Vincent Farines, Jean-Roch Mouret

**Affiliations:** ^1^SPO, University of Montpellier, INRAE, Institut Agro, Montpellier, France; ^2^UE Pech Rouge, INRAE, Gruissan, France; ^3^MISTEA, INRAE, Institut Agro, Montpellier, France

**Keywords:** alcoholic fermentation, wine, nitrogen additions, temperature, fermentative aromas, statistical modeling

## Abstract

Among the different compounds present in the must, nitrogen is an essential nutrient for the management of fermentation kinetics, also playing a major role in the synthesis of fermentative aromas. Fermentation temperature is yet another variable that affects fermentation duration and the production of fermentative aromas in wine. The main objective of this study was thus to evaluate the combined effects of nitrogen addition—at the start of the fermentation process or during the stationary phase—at different fermentation temperatures on both fermentation kinetics and aroma synthesis kinetics. To study the impact of these three parameters simultaneously, we used an innovative transdisciplinary approach associating an online GC-MS system with an original modeling approach: a Box-Behnken experimental design combined with response surface modeling and GAM modeling. Our results indicated that all three factors studied had significant effects on fermentation and aroma production kinetics. These parameters did not impact in the same way the different families of volatile compounds. At first, obtained data showed that reduction of ester accumulation in the liquid phase at high temperature was mainly due to important losses by evaporation but also to modifications of yeast metabolic capabilities to synthetize these compounds. In a noticeable way, optimal temperature changed for liquid accumulation of the two classes of esters—23°C for acetate ester and 18°C for ethyl esters—because biological impact of temperature was different for the two chemical families. Moreover, the study of these three factors simultaneously allowed us to show that propanol is not only a marker of the presence of assimilable nitrogen in the medium but above all a marker of cellular activity. Finally, this work enabled us to gain a deeper understanding of yeast metabolism regulation. It also underlines the possibility to refine the organoleptic profile of a wine by targeting the ideal combination of fermentation temperature with initial and added nitrogen concentrations. Such observation was particularly true for isoamyl acetate for which interactions between the three factors were very strong.

## 1. Introduction

The presence of fruity and floral aromas in the wine’s aromatic profile is increasingly sought after. This profile is composed of a set of volatile compounds belonging to different families of aromas, the main ones being higher alcohols, acetate esters and ethyl esters (Swiegers et al., 2005). For many years, numerous studies have been carried out to better understand the metabolism associated with the synthesis of these volatile compounds and to better manage their concentration during fermentation. Several environmental factors are known to influence this synthesis, in particular nitrogen and temperature (Bell and Henschke, 2005; Mouret et al., 2014b; Rollero et al., 2015).

Nitrogen is an essential nutrient for yeast growth and fermentation. A high nitrogen concentration in the must resulted in a high maximum fermentation rate along with a short fermentation time (Bely et al., 1990). Nitrogen also played an important role in the synthesis of aromas. Indeed, a high initial nitrogen concentration was associated with a high concentration of acetate esters (Hernandez-Orte et al., 2006; Ugliano et al., 2010; Torrea et al., 2011; Rollero et al., 2015; Seguinot et al., 2018). Conversely, the relationship between the synthesis of higher alcohols (such as isobutanol or isoamyl alcohol) and initial nitrogen content was not linear, with the exception of propanol. There was an optimum at about 200 mg/L of assimilable nitrogen and then the concentration of these alcohols decreased with nitrogen content (Jimenez-Marti et al., 2007; Carrau et al., 2008; Vilanova et al., 2012). Concerning propanol, it has been shown that this compound is a marker of assimilable nitrogen in the must as its concentration increased proportionally with initial nitrogen concentration (Mouret et al., 2014a).

To overcome nitrogen deficiencies in musts, nitrogen addition during fermentation has become a regular method. In addition to reducing fermentation duration, it promotes the synthesis of acetate esters and propanol (Seguinot et al., 2018; Godillot et al., 2022). Indeed, it was recently shown that nitrogen addition at the beginning of the stationary phase of fermentation activated the *ATF2* gene that codes for one of the isoenzymes responsible for the synthesis of acetate esters (Godillot et al., 2022). This overexpression resulted in maximizing the synthesis of this aromatic family. Regarding propanol, it was confirmed as a marker of must assimilable nitrogen since its concentration increased with the addition of nitrogen, regardless of the addition timing (Godillot et al., 2022).

Temperature has both a physical and a biological impact on the fermentation process. First, temperature impacted the length of the latency phase, the fermentation rate and thus the duration of fermentation (Bely et al., 1990). This parameter also affected the concentration of aroma compounds in the final wines (Torija et al., 2003; Morakul et al., 2011, 2013; Mouret et al., 2014a). A temperature increase induced the evaporation of all volatile compounds; but, for certain compounds—acetate and ethyl esters, this evaporation resulted in huge losses, because of their high volatility and hydrophobicity (Morakul et al., 2013). It was shown by Mouret et al. (2014a) that, during a fermentation carried out at 30°C, the loss by evaporation was, respectively, 30 and 50% for acetate esters and ethyl esters. With regard to the effect of temperature on the metabolism, it was noted that high temperatures induced the synthesis of higher alcohols while low temperatures induced the synthesis of ethyl esters (Beltran et al., 2006; Molina et al., 2007).

The objective of this work was therefore to study the cross-impact of three key parameters of the fermentation process—initial nitrogen, added nitrogen and temperature—on both fermentation kinetics and aroma production kinetics thanks to an innovative a multidisciplinary approach (online monitoring, bioprocess, modeling). At first, this methodology consists in the use of an online GC-MS system. Using this new device and a model predicting the evolution of the gas-liquid partitioning of aromas (Morakul et al., 2011; Mouret et al., 2014b), it was possible to perform mass balances of aroma production and to differentiate physical and biological impacts of fermentations parameters on aromas accumulation in the liquid phase. Then, to evaluate the impact of these three parameters simultaneously, two modeling approaches were implemented: a Box-Behnken design together with response surface modeling and a GAM modeling. This transdisciplinary approach will make it possible to have a global vision of the production kinetics of the aroma compounds during the alcoholic fermentation. Finally, from a practical point of view, the data obtained will allow us to evaluate if there is an optimal value for each of the three factors allowing to maximize the production of one or several target aromas.

## 2. Materials and methods

### 2.1. Yeast strain

The *Saccharomyces cerevisiae* strain used in this study was the commercial strain Lalvin EC1118^®^ (Lallemand SA, Montreal, QC, Canada). Fermentation tanks were inoculated with 10 g/hL active dry yeast previously rehydrated for 30 min at 37°C in a 50 g/L glucose solution.

### 2.2. Fermentation conditions

All fermentations were carried out in synthetic musts simulating a standard grape juice (Bely et al., 1990). The detailed compositions of the different stock solutions used for the preparation of this synthetic medium are identical to those presented in Mouret et al. (2014b). All musts contained 200 g/L of sugars (100 g/L glucose and 100 g/L fructose) and their pH was 3.3. Three initial assimilable nitrogen (composed of ammonium chloride and a mixture of amino acids) concentrations were tested: 70, 140, and 210 mgN/L, respectively, corresponding to media SM70, SM140, and SM210. The proportions of the different sources were identical in all three media.

Fermentations in SM70, SM140, and SM210 were run in 10-liter stainless steel tanks, at 18, 23, and 28°C. The amount of CO_2_ released was accurately and automatically measured with a gas mass flowmeter, for calculation of the rate of CO_2_ production (dCO_2_/dt).

Different concentrations—50, 100, and 150 mg N/L—of mineral nitrogen (di-ammonium phosphate) were added at 20 g/L of produced CO_2_ (corresponding to 24% of fermentation progress). Nitrogen additions were performed automatically using a peristaltic pump fed with the nitrogen solution. During fermentation, the total cell population was determined using a Beckman Coulter counter (Model Z2, Beckman-Coulter, Margency, France) fitted with a 100 μm aperture probe.

### 2.3. Online-based kinetic analysis of volatile compounds during the fermentation process

The concentrations of volatile compounds in the headspace of the fermentor were measured with an online GC-MS device.

We analyzed up to three aroma compounds from each major family of volatile compounds: isoamyl alcohol, isobutanol and propanol (higher alcohols), isoamyl acetate (acetate esters), ethyl hexanoate and ethyl octanoate (ethyl esters). The concentrations in the liquid phase were calculated as described by Morakul et al. (2011, 2013).

#### 2.3.1. Online measurements of concentrations in the gas phase

Headspace gas was pumped from the tank at a flow rate of 10 mL/min through a heated transfer line. Carbon compounds were concentrated in a cold trap (Tenax™) for 3 min, desorbed at 310°C for 1 min, and analyzed with a GC Thermo TRACE 1300 (Thermo Fisher Scientific™, Toulouse, France) dedicated to the separation of carbon compounds. The column output was then led to two analyzers: an FID detector and an MS Thermo ISQ7000 (Thermo Fisher Scientific™, Toulouse, France).

The GC oven program was as follows: 38°C for 7 min, increase to 65°C at a rate of 3°C/min followed by an increase to 160°C at a rate of 6°C/min followed by an increase to 210°C at a rate of 8°C/min and a final hold at 210°C for 4 min. The peaks areas were acquired with Chromeleon Chromatography Data System (CDS) Software (Thermo Fisher Scientific™, Toulouse, France).

The calibration of the instrument was done using an ATIS system (Adsorbent Tube Injector System; Supelco, Bellefonte, PA, USA) which allowed to produce standard gases by vaporizing a liquid reference sample in a continuous flow of inert gas. First, a calibration range must be prepared and injected (5 μL) into the ampoule. The latter was continuously swept by a hot air flow at 30 mL/min. The concentration range—in μg/L of gas—was: 0.88–36.48, 0.40–16.58, 0.24–9.87, 1.02–43.43, 0.24–10.19, 0.29–12.43 for isoamyl alcohol, isobutanol, propanol, isoamyl acetate, ethyl hexanoate and ethyl octanoate, respectively.

Concentrations in the liquid, losses and total production were calculated using a custom R version 3.6.2 (R Core Team, 2022) program from the concentration measured online in the gas phase. Details of these calculations are presented in the following parts.

#### 2.3.2. Calculation of the concentration in the liquid

The concentration of a volatile compound in the liquid [*C^liq^*(*t*)] was calculated from the concentration measured online in the gas phase, expressed as *C^gas^*(*t*) in mg/L of CO_2_, using the partition coefficient (*k_i_*) value [Eq. (1)].


(1) $$C^{l\,i\,q}\,\left(t\right)=\frac{C^{g\,a\,s}\,\left(t\right)}{k_{i}}$$

The value of *k_i_* [Eq. (2)] was calculated with the model developed by Morakul et al. (2011), as a function of the fermenting must composition, characterized by ethanol concentration, and temperature.


(2) $$l\,n\,k_{i}=F\,1+F\,2*E-\frac{F\,3+F\,4*E}{R}\,\left(\frac{1000}{T}-\frac{1000}{T\,r\,e\,f}\right)$$

where *E* is the ethanol concentration (g/L) in the liquid phase, calculated from measurements of the amount of CO_2_ released; *T* is the current absolute temperature, *Tref* corresponds to the absolute reference temperature (i.e., 293.15 K, 20°C, in this study). *F1*, *F2*, *F3*, and *F4* are constants, identified for each volatile compound. The values of these parameters (Table 1) for the different molecules were determined by Mouret et al. (2014a,b).

**TABLE 1:** Experimental conditions for the 15 fermentations.

Experiments	Initial nitrogen (N0), mg/L	Nitrogen added (Nad), mg/L	Temperature (Temp), °C
1	70	50	23
2	210	50	23
3	70	150	23
4	210	150	23
5	70	100	18
6	210	100	18
7	70	100	28
8	210	100	28
9	140	50	18
10	140	150	18
11	140	50	28
12	140	150	28
13^1^	140	100	23
14^2^	140	100	23
15^3^	140	100	23

^1–3^Three replicates included at the center of the experimental domain.

#### 2.3.3. Calculation of the losses in the exhaust gas

The losses in the gas were calculated with Eq. (3).


(3) $$L\,\left(t\right)=\int_{0}^{t}C^{g\,a\,s}\,\left(t\right)*Q\,\left(t\right)*dt$$

where *Q*(*t*) is the CO_2_ flow rate at time t, expressed in liters of CO_2_ per liter of must and per hour. The relative losses (*RL*), expressed as a percentage of production [*P*(*t*)], were determined as follows [Eq. (4)]:


(4) $$R\,L=\frac{L\,\left(t\right)}{P\,\left(t\right)}=\frac{\int_{0}^{t_{e\,n\,d}}C^{g\,a\,s}\,\left(t\right)*Q\,\left(t\right)*dt}{C^{l\,i\,q}\,\left(t_{e\,n\,d}\right)+\int_{0}^{t_{e\,n\,d}}C^{g\,a\,s}\,\left(t\right)*Q\,\left(t\right)*dt}$$

where *t*_*end*_ corresponds to the end of fermentation (h).

#### 2.3.4. Calculation of the total production

The total production of a volatile compound at time t, expressed as *P*(*t*) in mg/L of must, was calculated by adding the concentration in the liquid phase, expressed as *C^liq^*(*t*) in mg/L of must, to the amount of the volatile compound lost in the gas phase, expressed as *L*(*t*) in mg/L of must [Eq. (5)].


(5) $$P\,\left(t\right)=C^{l\,i\,q}\,\left(t\right)+L\,\left(t\right)$$

This total production reflects yeast ability to produce a volatile compound, regardless of its subsequent fate: accumulation in the liquid phase or evaporation.

#### 2.3.5. Calculation of total production rates

The calculation of total production rates was performed using the first derivative of a local regression [using the *locfit(*) R function] performed on the total production (mg/L). These rates were calculated only on propanol to further understand the effects of the three factors studied in this work on its production kinetics.

### 2.4. Statistical analyses

The data set generated in this study is substantial. To analyze the impact of the three factors studied in the present work on the production of volatile compounds and to answer various biological questions, extensive work on the data was performed in three steps. The first step consisted in a smoothing realized on production kinetics in order to work on a clean data set. The second step used a reliable method to calculate the three following concentrations—liquid concentration, losses and total production; it was implemented in the same way for all fermentations. The third step involved different statistical analyses used to answer the different biological questions: (i) a GAM modeling to investigate the impact of factors on the whole shape of the production kinetics and (ii) a statistical analysis of the Box-Behnken design to study the impact of factors on the final production of volatile compounds. At last, the total propanol production rate was calculated.

Statistical analysis was performed with R software, version 4.0.2 (R Core Team, 2022) and the rsm library (Lenth, 2009) for the Box-Behnken design, the mgcv library (Wood, 2017) for GAM modeling and the tidyverse library (Wickham et al., 2019) for data management and plot purposes.

#### 2.4.1. Experimental design

A Box-Behnken experimental design was applied to investigate the effects of three independent variables (initial nitrogen, concentration of nitrogen addition, and temperature) and to calculate the optimal combination for fermentation kinetics and the different final concentrations of volatile compounds, with a limited number of experimental runs. A total of 15 fermentations were performed, including triplicates at the center of the experimental domain (Table 1).

In this experimental design, there were three coded factor levels for each factor: −1, 0, +1 in which −1 corresponded to the low level, +1 to the high level, and 0 to the mid-level. The actual level of each factor was calculated with the following equation (Eq. 6) (Box and Behnken, 1960):


(6) Actuallevel=Codedvalue×

$$\frac{H\,i\,g\,h\,v\,a\,l\,u\,e-L\,o\,w\,v\,a\,l\,u\,e}{2}+\frac{H\,i\,g\,h\,l\,e\,v\,e\,l+L\,o\,w\,l\,e\,v\,e\,l}{2}$$

#### 2.4.2. GAM modeling

The curves of fermentation kinetics and production kinetics of volatile compounds have been modeled by Generalized Additive Models (GAMs; Wood, 2017). Indeed, each fermentation produces complex non-linear curves, and GAMs are much more flexible than either classical linear models or generalized linear models. Hierarchical GAMs (Pedersen et al., 2019) were used: these models allow smooth functional relationships between a predictor and a response to vary between groups, in such a way that the different functions are in some sense pooled toward a common shape. Indeed, we want to know how the functional relationships vary between groups, and whether a relationship holds across groups. Several models can be used to answer these questions, and we decided to use a ≪ GS model ≫ by Pedersen et al. (2019). In such a model, the relationship between a predictor x and a response Y is modeled by the sum of a global smoother and group-level smoothers. Each group-level smoother is associated to a level of one grouping factor in the model.

In this work, several responses were studied. First, to study fermentation kinetics, the CO_2_ flow rate was modeled as a function of consumed sugar: response Y is the CO_2_ flow rate and predictor x is consumed sugar. Then, to study the production kinetics of aromas, the concentration in the liquid, the losses and the total production of volatile compounds were modeled as functions of released CO_2_: Y is *C^liq^*, *L*, or *P*, and x is released CO_2_. The volatile compounds studied were propanol, ethyl hexanoate, ethyl octanoate, isoamyl acetate, isoamyl alcohol, isobutanol, and also the ratio isoamyl acetate to isoamyl alcohol.

Two types of GS model were used:

For the first type of model (GS1), only one grouping factor in the model that corresponds to the observed combination of T°C, N0 and Nadd is used. Table 1 shows that 15 fermentations were performed, corresponding to 13 combinations of the three factors (one combination is repeated three times). If we denote by i the combination of the three factors and j the repetition, the formula of this model is given by:

Model GS1 (eq. 7)


(7) $$Y_{i\,j}=f\,\left(x_{i\,j}\right)+f_{i}\,\left(x_{i\,j}\right)+\in_{i\,j}$$

W⁢i⁢t⁢h⁢i=1,…,13;j=1,2,3

Where *f* is the global smoother and *f_i_*the smoother specific to the *i*th combination of the three factors. The global function should be viewed as a useful summary of the average trend across the groups, that is as a mean trend. The *i*th group-specific function represents group specific deviation from the global function.

For the second type of model (GS2), we have three grouping factors with three levels each (T°C, N0, Nadd) and their interactions with 9 levels each (Temp × N0, Temp × Nadd, and Nadd × N0). If we denote by *i* the level of Temp, *j* the level of N0, *k* the level of Nadd and *l* the repetition, the formula of this model is given by:

Model GS2 (Eq. 8):


(8) $$Y_{i\,j\,k\,l}=f\,\left(x_{i\,j\,k\,l}\right)+f_{i}\,\left(x_{i\,j\,k\,l}\right)+f_{j}\,\left(x_{i\,j\,k\,l}\right)+f_{k}\,\left(x_{i\,j\,k\,l}\right)$$

$$+f_{i\,j}\,\left(x_{i\,j\,k\,l}\right)+f_{i\,k}\,\left(x_{i\,j\,k\,l}\right)+f_{j\,k}\,\left(x_{i\,j\,k\,l}\right)$$

$$+,\in_{i\,j\,k\,l}$$

W⁢i⁢t⁢h⁢i=18,23,28;j=70,140,210;

k=50,100,150;l⁢1,2,3

Where *f* is the global smoother, *f_i_* a smoother specific for the *i*th level of Temp, *f_j_* a smoother specific for the *j*th level of N0, *f_k_* a smoother specific for the *k* th level of Nadd, *f*_*ij*_ a smoother specific for the interaction level *i* *j between Temp and N0, *f*_*ik*_ a smoother specific for the interaction level *i* *k between Temp and Nadd and *f*_*jk*_ a smoother specific for the interaction level *j* *k between N0 and Nadd.

In both models GS1 and GS2, a global smoother represents the average trend across the groups. The main difference between them is that in GS1, only one grouping factor which is the combination of Temp, N0 and Nadd is considered whereas in GS2, this grouping factor is decomposed into three grouping factors (Temp, N0, Nadd) and their interactions. Hence, if only one group-specific function is considered in GS1, 6 group-specific functions are considered for GS2. Compared to GS2, the GS1 model is simpler and the figures obtained are easier to understand. However, model GS2 enables us to decompose the group-specific function, to understand which ones of the factors and/or interactions are relevant to explain the deviations from the average trend.

#### 2.4.3. Response surface

The effect of the three independent variables on each measured parameter (Y) was modeled using a polynomial response surface (Eq. 9):


(9) $$Y=\beta_{0}+\beta_{1}\,x_{1}+\beta_{2}\,x_{2}+\beta_{3}\,x_{3}$$

$$+\beta_{12}\,x_{1}\,x_{2}+\beta_{13}\,x_{1}\,x_{3}+\beta_{23}\,x_{2}\,x_{3}$$

$$+\beta_{11}\,x_{1}^{2}+\beta_{22}\,x_{2}^{2}+\beta_{33}\,x_{3}^{2}+\varepsilon$$

where *x_1_*, *x_2_* and *x_3_* represent the coded values of initial nitrogen content, concentration of nitrogen added and fermentation temperature, respectively, *Y* is the predicted response (fermentative parameters or final concentration of volatile compounds), β_0_ the intercept term, β_*i*_ the coefficients associated to the linear terms, β_*ii*_ the coefficients associated to the quadratic terms, and β_*ij*_ the coefficients associated to the interaction terms (*i*=1, 2, and 3; *j*=1, 2, and 3) and ε are independent N (0, σ^2^) distributed error terms. A simplified model was fitted for some compounds by suppressing the interactive terms of the equation according to validity criteria. The normality of residual distributions and homogeneity of variance were studied with standard diagnostic graphs; no violation of the assumptions was detected. The accuracy and general ability of each polynomial model described above were evaluated by a lack-of-fit test, the Fisher test and the adjusted coefficient of determination adjR^2^. The reliability of the fitted models was overall very good: each polynomial model gave a non-significant lack-of-fit test at a 0.05 threshold, a significant Fisher test at a 0.05 threshold and an adjR^2^ range between 70 and 93%. For graphical representations of the surface responses, a custom version of the persp() function was used.

## 3. Results

### 3.1. GAM models

Generalized Additive Model modeling with GS1 and GS2 was applied to evaluate the cross-impact of the three factors on kinetics shape (main fermentation reaction and aroma compounds). The two models were consistent with each other and complementary. They provided remarkably close results in terms of predictions and AIC (Akaike Information Criterion), so both of them were used to interpret our results. The first model (GS1) analyzed the effect of the modalities (combination of the different levels of the factors) in relation to the average curve while the second model (GS2) analyzed the impact of each factor (with its different levels) and the potential interactions existing between the studied factors. The latter allowed us to determine which factor most influenced (positively or negatively) kinetics shape. Moreover, model GS1 was used to present the figures and model GS2 to obtain *p*-values for determining the significance of the smoothers specific to Temp, N0, Nadd and their interactions.

#### 3.1.1. Fermentation kinetics

**FIGURE 1:**
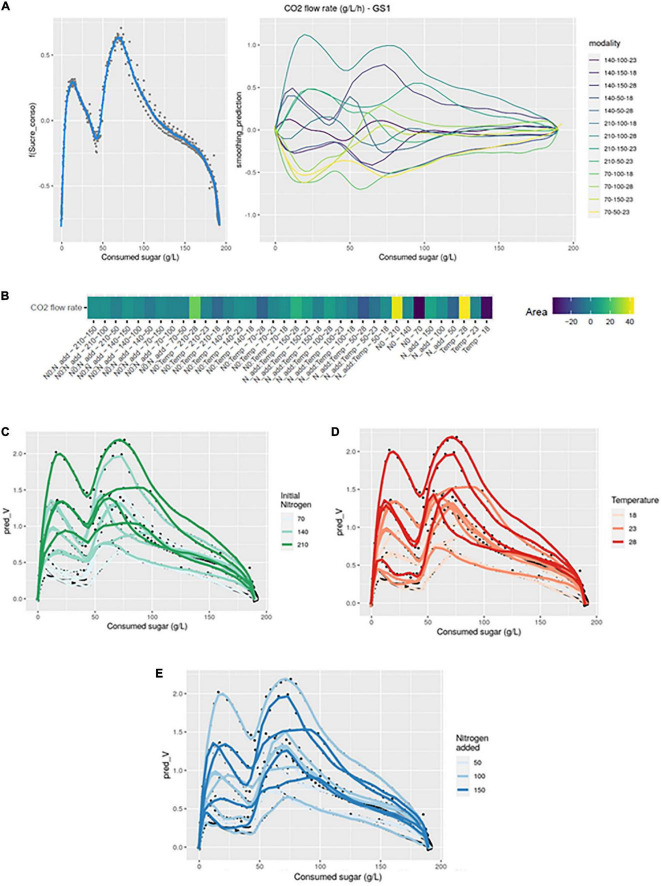
GAM modeling of fermentation kinetics. **(A)** Left: Smoothing average of the fermentation rate, right: Deviations of each modality from this average smoothing. **(B)** Heatmap representing magnitudes of each factor and every possible interaction using areas under the curves of the deviations from the average smoothing. **(C–E)** Prediction curves for each modality represented with a color depending on the level of NO, temperature and Nadd.

In the GS1 model (Figure 1A), the left graph represented the average smoothing of fermentation rate: it was an average of all curves. The right graph represented the deviations of each curve from this average smoothing. A prediction curve can be retrieved by summing the average smoothing curve with the corresponding deviation curve.

The average smoothing curve was characterized by a first maximal fermentation rate (Vmax1), followed by a second maximal value (Vmax2). Looking at the deviations from the average smoothing, we can see that the first and the second Vmax were visible, sometimes in positive and sometimes in negative values. Positive values meaned that the Vmax of the corresponding curve was higher than the average Vmax, while negative values meaned that the Vmax of the corresponding curve was lower than the average Vmax. For instance, the higher deviation was the one corresponding to the curve 210-100-28, meaning that this was the experiment with the two larger Vmax. On the opposite, the curve 70-100-18 had large negative values, meaning that this was the experiment with the two lower Vmax. In the middle of all curves, one with very small deviations was the 140-100-23, meaning that this experiment had Vmax close to the average smoothing ones.

The heatmap (Figure 1B) summarized the different effects of the GS2 model. The magnitudes of each factor and every possible interaction were represented by the area under the corresponding curves of the deviations from the average smoothing (plots not shown but available in the online repositories). For instance, the area under the estimated curve *f_i_* represented the magnitude of the temperature, the area under the estimated curve *f_j_* represented the magnitude of the initial nitrogen and the area under the estimated curve *f*_*ij*_ represented the magnitude of the interaction between temperature and initial nitrogen. The magnitudes for each single effect and every possible interaction were represented on a heatmap (Figure 1B), using a gradient of colors. In this figure the factors were ranked according to their magnitude and we can easily identify the factors with the greatest influences on the studied production curve. We can note that, using statistical tests, all simple effects and all interactions terms were significant.

In Figures 1C–E we focused on the simple effects of initial nitrogen, temperature and added nitrogen. Indeed, the prediction curves for each modality were represented with a color depending on the level of initial nitrogen, temperature and added nitrogen, respectively. On Figure 1C, we can see that high fermentation rates were associated with large initial values for nitrogen. On Figure 1D, we can see that high fermentation rates were associated with large temperatures as well. Figure 1E was more difficult to interpret because there were interactions between Nadd and temperature, and Nadd and N0. Such a result indicated that the effect of Nadd depended on the values of N0 and temperature; its impact was higher if the initial nitrogen concentration and temperature were low.

#### 3.1.2. Volatile compounds

As for fermentation kinetics, GAM modeling was applied to investigate the effects of factors on fermentative aroma production kinetics. All kinetics of the different volatile compounds studied were significantly impacted by the three factors N0, Nadd and Temp. However, it was necessary to determine the importance of each factor on the course of aromatic kinetics in order to better understand their impact. The modeling was applied to both liquid phase concentration and total production for isoamyl acetate and ethyl esters. Only liquid phase concentration was analyzed for higher alcohols as these compounds being weakly volatile, their losses by evaporation are negligible.

##### 3.1.2.1. Isoamyl alcohol

**FIGURE 2:**
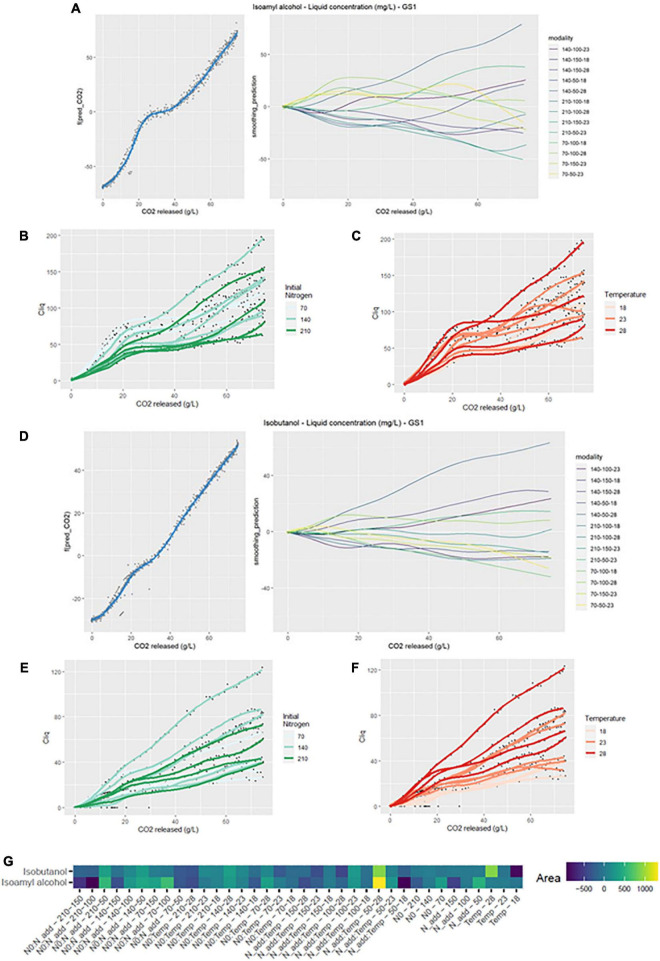
GAM modeling of ISOH and ISB production kinetics. **(A,D)** Left: Smoothing average of ISOH and ISB concentration, right: Deviations of each modality from this average smoothing. **(B,C,E,F)** Prediction curves for each modality represented with a color depending on the level of NO and temperature. **(G)** Heatmap representing magnitudes of each factor and every possible interaction using areas under the curves of the deviations from the average smoothing, for ISOH and ISB.

For isoamyl alcohol production, the average smoothing curve of the modalities showed two distinct phases (Figure 2A): a first phase was observed before nitrogen addition; then, after addition, a change of slope seemed to tend toward a decrease in isoamyl alcohol production. Furthermore, the deviation graph indicated that differences from the mean were observed both before and after nitrogen addition.

In Figure 2G, we can see that the interaction effects N0:Nadd (210–150) and Nadd:Temp (50–18) corresponded to large area under the corresponding curves in model GS2, meaning that they had high impact on kinetics shape. In particular, these modalities showed negative divergences from the average smoothing curve. On the other hand, a significant positive divergence from the average smoothing was found for the interaction effect Nadd:Temp (50–28). Regarding the simple effects, the kinetics of isoamyl alcohol was mostly impacted by initial and added nitrogen amounts. When the value of these factors increased, the sign of divergence tended to turn negative.

The effect of initial nitrogen can also be seen in Figure 2B. The production of isoamyl alcohol tended to be larger with small values of initial nitrogen concentration. Nevertheless, the highest final liquid phase content was reached when N0 is at 140 mg/L (Table 2). This observation is in agreement with previous studies (Mouret et al., 2014a; Rollero et al., 2018; Seguinot et al., 2018), which noted that the optimal concentration for isoamyl alcohol production appears to be around 140 mg/L of initial assimilable nitrogen.

**TABLE 2:** Group averages (N0, Nadd and Temp) of final aroma concentrations in the liquid (top table) and for total production (bottom table).

	(IsoA) mg/L	(±)	(IsoOH) mg/L	(±)	(IsbOH) mg/L	(±)	(EtH) mg/L	(±)	(EtO) mg/L	(±)	(Prop) mg/L	(±)	Ratio IsoA (mol)	(±)
MS210	1.53	0.4	97.14	36.7	50.74	15.1	0.341	0.11	0.336	0.13	49.55	8.9	0.0113	0.004
MS140	1.23	0.28	124.53	31.9	72.72	26.6	0.301	0.07	0.29	0.13	50.35	12.7	0.0071	0.003
MS70	0.98	0.4	107.99	11.2	40.93	15.7	0.314	0.1	0.247	0.14	40.28	14.3	0.0064	0.003
18°C	1.15	0.45	105.58	17.3	35.22	6.52	0.42	0.03	0.429	0.033	43.71	13.7	0.0078	0.004
23°C	1.42	0.39	116.88	27	59.73	19.7	0.316	0.04	0.267	0.114	45.24	12.7	0.0088	0.004
28°C	1.03	0.15	112.93	48.3	79.18	27.1	0.209	0.02	0.195	0.099	55.06	9.5	0.007	0.003
50N	1.02	0.52	142.77	28.2	65.78	36.3	0.331	0.07	0.388	0.108	33.64	10.5	0.0048	0.002
100N	1.25	0.33	112.3	22.2	58.64	20.9	0.307	0.11	0.244	0.134	49.66	6.1	0.0077	0.002
150N	1.46	0.22	83.75	9.2	50.52	22.5	0.313	0.09	0.275	0.102	57.4	11	0.012	0.003
MS210	1.94	0.42	97.96	36.91	51.07	15.2	0.568	0.12	0.562	0.18	49.8	8.963	0.0143	0.005
MS140	1.57	0.35	125.63	32.32	73.19	26.9	0.482	0.06	0.472	0.16	50.6	12.8	0.009	0.003
MS70	1.22	0.47	109.2	11.41	41.26	15.9	0.494	0.1	0.393	0.18	40.5	14.41	0.0079	0.004
18°C	1.32	0.51	106.16	17.31	35.36	6.5	0.578	0.05	0.595	0.028	43.84	13.68	0.0089	0.004
23°C	1.78	0.49	117.93	27.1	60.09	19.8	0.52	0.08	0.456	0.189	45.44	12.7	0.011	0.005
28°;C	1.47	0.23	114.47	48.83	79.89	27.4	0.417	0.05	0.389	0.187	55.36	9.545	0.0098	0.004
50N	1.36	0.71	144.05	28.74	66.25	36.7	0.546	0.09	0.632	0.144	33.8	10.59	0.0062	0.003
100N	1.56	0.36	113.35	22.4	59.02	21	0.488	0.1	0.397	0.172	49.87	6.169	0.0096	0.003
150N	1.84	0.28	84.6	9.26	50.87	22.7	0.505	0.07	0.456	0.097	57.63	11.1	0.015	0.004

##### 3.1.2.2. Isobutanol

Concerning the kinetics of isobutanol production, the GS model led to an aromatic signature almost similar to that of isoamyl alcohol (Figure 2D). Divergences from the smoothing average were already visible before nitrogen addition, but increased substantially after the addition.

In contrast to isoamyl alcohol, the presence of interaction effects on the heatmap was less important (Figure 2G). Simple Temp effect (28) and interaction Nadd:Temp effect (50–28) showed a significant positive divergence from the smoothing average. It also appeared that temperature had a substantial influence on the kinetics of this compound, a gradient was visible on Figure 2F. Finally, a concentration of 140 mg/L seemed to be optimal for isobutanol production (Figure 2E and Table 2).

##### 3.1.2.3. Isoamyl acetate

**FIGURE 3:**
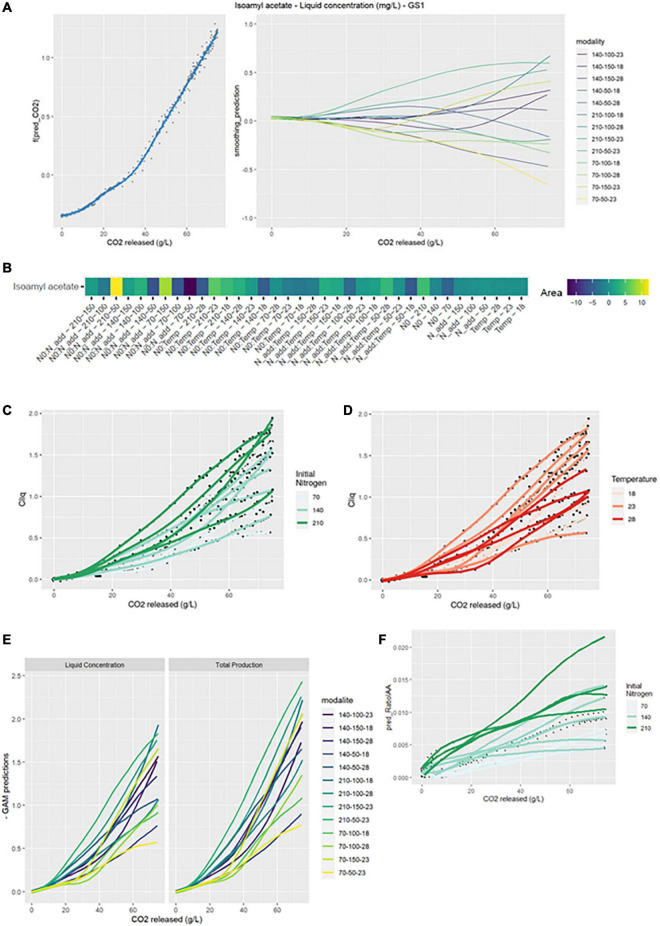
GAM modeling of IAA production kinetics. **(A)** Left: Smoothing average of IAA concentration, right: Deviations of each modality from this average smoothing. **(B)** Heatmap representing magnitudes of each factor and every possible interaction using areas under the curves of the deviations from the average smoothing. **(C,D)** Prediction curves for each modality represented with a color depending on the level of NO and temperature. **(E)** Comparison between liquid concentration and total production. **(F)** Predicted ratio of lAA to lSOH vs. CO_2_ released, with a color depending on the level of NO.

For the production of isoamyl acetate, the smoothing average of the modalities was mainly impacted by the addition of nitrogen (GS1 model). Indeed, a change in slope was observed following the addition that accelerated the production of this compound (Figure 3A). All divergences from the smoothing average were observed after nitrogen addition.

The heatmap (GS2 model) highlighted numerous interaction effects influencing the shape of the curves. Indeed, N0:Nadd, N0:Temp and Nadd:Temp effects all had a significant impact. The largest positive divergences from the smoothing average were observed for the N0:Nadd-210-50 and N0:Nadd-70-150 combinations. Conversely, the largest negative divergences were observed for the combinations N0:Nadd-70-50, N0:Temp-210-28, or Nadd:Temp-50-18.

Initial nitrogen seemed to promote the production of isoamyl acetate in the liquid phase (Figure 3C). Thus, average concentrations of 1.53 and 0.98 mg/L were obtained for an initial nitrogen of 210 and 70 mg/L, respectively. Similarly, a high concentration of added nitrogen increased the liquid phase concentration in isoamyl acetate: an average of 1.46 mg/L was obtained for an addition of 150 vs. 1.02 mg/L for an addition of 50 mg/L (Table 2). Conversely, the temperature effect on the concentration of isoamyl acetate seemed to reach an optimum at 23°C (Figure 3D). The highest final concentration in the liquid phase was obtained at 23°C (1.42 mg/L) compared to 18°C (1.15 mg/L) or 28°C (1.03 mg/L) (Table 2).

The comparison between the concentration in the liquid phase and total production showed that the evaporation of the compound at high temperatures was substantial (Figure 3E): evaporation losses for the 28°C modalities were about 30 vs. 14% for the 18°C modalities (Table 2).

Total production and the concentration in the liquid were impacted by the same effects (Table 2), indicating that the impact of temperature is both biological and physical.

##### 3.1.2.4. Ratio IAA/ISOH

In order to evidence the impact of the three different factors on the conversion of isoamyl alcohol into isoamyl acetate, the ratio between the total production of these two compounds was calculated and then modeled by GAM. The shape of this ratio was significantly impacted by initial and added nitrogen amounts. As already observed by Godillot et al. (2022), a high concentration of initial and added nitrogen will increase the ratio and thus the conversion of isoamyl alcohol to isoamyl acetate. For an initial nitrogen concentration of 210 mg/L, a ratio of 0.0143 was obtained compared to 0.0079 with 70 mg/L of nitrogen. A high concentration of added nitrogen doubled the ratio value compared to a low concentration addition (Table 2 and Figure 3F). Moreover, the ideal temperature for the conversion of alcohol to isoamyl acetate seemed to be 23°C, in agreement with the results previously shown for isoamyl acetate (Table 2).

##### 3.1.2.5. Ethyl hexanoate

**FIGURE 4:**
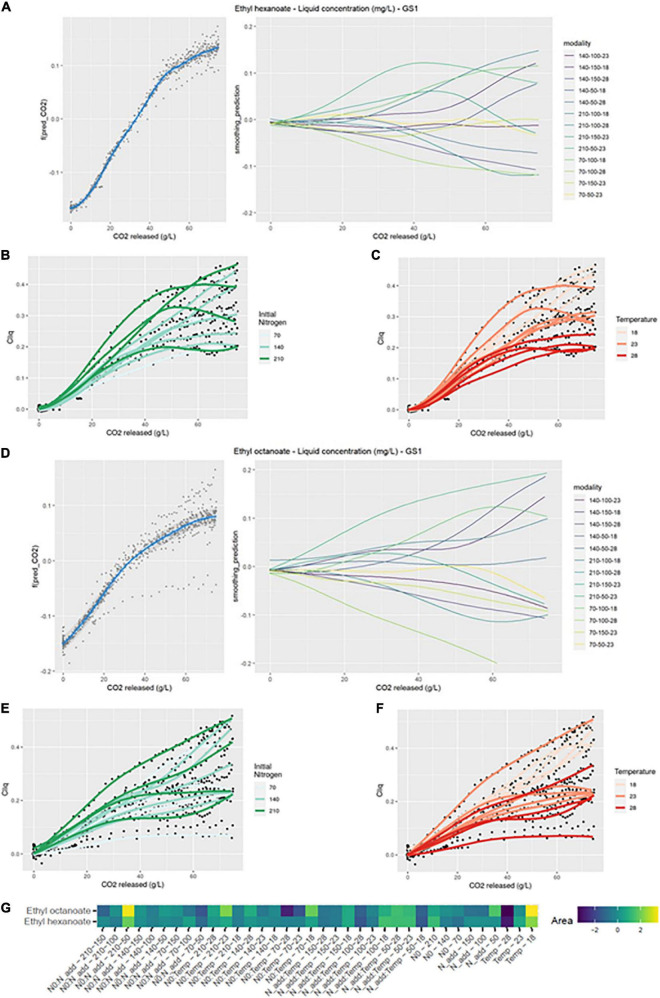
GAM modeling of EHE and EOC production kinetics. **(A,D)** Left: Smoothing average of EHE and EOC concentration, right: Deviations of each modality from this average smoothing. **(B,C,E,F)** Prediction curves for each modality represented with a color depending on the level of NO and temperature. **(G)** Heatmap representing magnitudes of each factor and every possible interaction using areas under the curves of the deviations from the average smoothing, for EHE and EOC.

As with isoamyl acetate, the production kinetics of ethyl hexanoate in the liquid phase was significantly impacted by interaction effects between the three factors (Figure 4G). The largest positive divergences from the smoothing average were for N0:Nadd-210-50, N0:Temp-70-18, and N0:Temp-210-18. Whatever the initial nitrogen concentration or the dose of added nitrogen, low temperatures positively influenced the production kinetics of this compound (Table 2 and Figures 4B, C). This result was supported by the simple effect of temperature on the kinetics. Indeed, low temperature resulted in positive value of the divergences (Figure 4G). This result can be related to an important evaporation of this compounds linked to its high hydrophobicity. Consequently, up to 60% of the produced ethyl hexanoate were lost for the fermentation modalities at 28°C, as reported in a previous study (Mouret et al., 2014a).

Ethyl hexanoate total production as well as the concentration in the liquid were impacted by the same effects (Table 2). The simple effect of the temperature was again found, indicating that temperature was the main factor that influences the kinetics. Synthesis of ethyl hexanoate by yeast was enhanced at temperatures lower than or equal to 23°C. The temperature not only affected the evaporation of this compound but also its synthesis by yeast.

##### 3.1.2.6. Ethyl octanoate

The signature of ethyl octanoate in the liquid differed from that of ethyl hexanoate (Figure 4D left). Divergences from the smoothing average were visible before nitrogen addition and they increased until the end of fermentation.

On the heatmap, the production kinetics of ethyl octanoate displayed the same interaction effects as for ethyl hexanoate and the factor with the largest divergence from the mean was the temperature (Figure 4G).

The liquid concentration of ethyl octanoate was impacted, like ethyl hexanoate, by the interactions between the three factors (Figure 4G). The initial nitrogen concentration clearly promoted ethyl octanoate production; the average concentration increased from 0.24 to 0.34 mg/L between low and high concentration of initial nitrogen (Table 2). Like for ethyl hexanoate, the negative effect of temperature was noticeable (Figure 4F). On average, the concentration of ethyl octanoate was divided by two when the temperature varied from 18 to 28°C. As for ethyl hexanoate, evaporation losses reached 60% for some modalities at 28°C (Table 2). Finally, added nitrogen did not seem to increase the final concentration of the compound (Table 2).

The effects of the factors on total ethyl octanoate production were the same as for the liquid concentration of this compound (Table 2). This last result indicated that the impact of temperature on ethyl esters accumulation in the liquid was both biological and physical.

##### 3.1.2.7. Propanol

**FIGURE 5:**
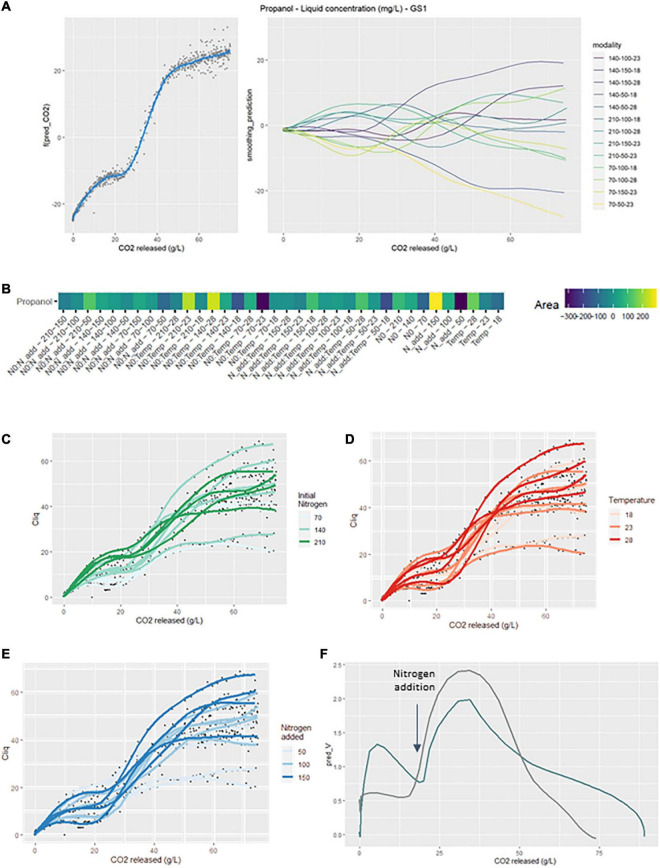
GAM modeling of propanol production kinetics. **(A)** Left: Smoothing average of the propanol concentration, right: Deviations of each modality from this average smoothing. **(B)** Heatmap representing magnitudes of each factor and every possible interaction using areas under the curves of the deviations from the average smoothing. **(C–E)** Prediction curves for each modality represented with a color depending on the level of NO, temperature and Nadd. **(F)** Rate of released CO_2_ (dark blue) and rate of total propanol production (gray) vs. the CO_2_ released.

The concentration of propanol in the liquid depended on the three factors studied and their interactions. The signature of propanol was particularly sensitive to the addition of nitrogen, since an acceleration of its production was observed after nitrogen addition (Figure 5A right). Moreover, divergences from the smoothing average were visible before nitrogen addition, depending on the initial nitrogen. There were larger divergences after nitrogen addition suggesting that the effect of added nitrogen was greater than that of initial nitrogen.

The GS2 model (Figure 5B) indicated that propanol production kinetics were positively impacted by N0:Temp-210-23 and N0:Temp-140-28. Conversely, the modalities N0:Temp-140-18 and N0:Temp-70-23 exhibited negative divergences from the smoothing average. The interaction between initial nitrogen and temperature strongly influenced the course of the production kinetics of this compound. The simple effect of added nitrogen showed significant positive divergences when the factor concentration was high.

The effect of the initial nitrogen concentration was clearly noticeable before 20 g/L of released CO_2_ since propanol concentration increased with the initial nitrogen content (Figure 5C). This result was consistent with those found in previous studies (Mouret et al., 2014b; Rollero et al., 2015; Seguinot et al., 2018). Similarly, for added nitrogen, propanol concentration increased both with the added nitrogen dose (Figure 5E) and with fermentation temperature (Figure 5D).

Total propanol production rate for the smoothing average was calculated in an attempt to understand the production dynamics of this specific compound. This production rate was compared to the CO_2_ release rate: total propanol production rate was found directly related to fermentation kinetics (Figure 5F).

### 3.2. Box-Behnken analysis

A Box-Behnken design was used to study the sign and weight of the three factors on some key parameters of the fermentation kinetics and on the final aroma contents. This statistical analysis is complementary to the GAM modeling done previously. The results of the simple, interaction and quadratic effects are summarized in Table 3.

**TABLE 3:** Box-Behnken effects on final volatile compounds production.

Para- meters	Simple effects			Interactions			Quadratic effects		
	**N0**	**Nad**	**T**	**N0: Nad**	**Nad: T**	**N0: T**	**N0**	**Nad**	**T**
Tf	−	−	−			+	+		
Lagphase			−			+			+
dVmax	−	+	+	−	+	−		−	
Isoamyl alcohol		−						−	
Isobutanol			+				−		
Propanol		+	+						
Isoamyl acetate	+	+		−					−
Ethyl hexanoate			−						
Ethyl octanoate			−					−	

#### 3.2.1. Parameters of fermentation kinetics

Three parameters, calculated as previously described by Godillot et al. (2022), were studied to summarize the fermentation kinetics: fermentation time (Tf), lag phase (Lagphase) and the difference between the first and second Vmax (dVmax). Fermentation time was significantly impacted by all three factors. Initial and added nitrogen contents had a negative effect as they reduced fermentation time. Moreover, increasing temperature also reduced fermentation time (Torija et al., 2003; Sablayrolles, 2009). The lag phase depends on the temperature (Colombie et al., 2005; Rollero et al., 2015): a high temperature shortens the lag time. Finally, the difference in Vmax was significantly impacted by all three factors. Initial nitrogen and temperature increased the difference in Vmax while the opposite was observed for added nitrogen in agreement with previous studies (Malherbe et al., 2004; Godillot et al., 2022).

#### 3.2.2. Isoamyl alcohol, isobutanol, and propanol

The final production of higher alcohols was differently impacted by the three factors studied in the present work. For isobutanol and propanol, a positive effect of the temperature was noticed. This result matches with the results found after GAM analysis. Furthermore, a negative effect of added nitrogen for isoamyl alcohol supports previous results (Seguinot et al., 2018; Godillot et al., 2022). Finally, a positive effect of added nitrogen on propanol confirms the results of GAM and previous studies (Mouret et al., 2014a; Seguinot et al., 2018; Godillot et al., 2022). These results imply that the management of the synthesis of these three higher alcohols is different.

#### 3.2.3. Isoamyl acetate

For isoamyl acetate, the effects of initial nitrogen, added nitrogen and temperature on the final concentration were the same for liquid phase concentration and total production. Initial and added nitrogen had a significant positive effect on this ester. These results confirm those found in previous studies (Beltran et al., 2005; Seguinot et al., 2018). The effect of temperature on this ester is in agreement with the results found in the GAM analysis and the quadratic effect of temperature shows an optimum at 23°C.

#### 3.2.4. Ratio IAA/IAOH

For the ratio of higher alcohols conversion to acetate esters, the effects of the factors were identical between the concentration in the liquid and the total production. There was no significant effect of temperature. On the opposite, significant and positive effects of both initial and added nitrogen were observed, supporting the results found in a previous study (Godillot et al., 2022). High initial or added quantities of nitrogen both results in a greater conversion of isoamyl alcohol to isoamyl acetate. This result is also in line with those found by GAM analysis.

#### 3.2.5. Ethyl hexanoate and octanoate

Added nitrogen had a significant negative effect on the concentration of ethyl octanoate in the liquid. Temperature also had a significant negative effect on both esters for both liquid concentration and total production. This result is consistent with the GAM analysis but also with Mouret et al. (2014b) that brought out a strong evaporation of esters (50–60%) but also a decrease in their synthesis by the yeast when the fermentation temperature increases.

## 4. Discussion

The main originality of the present work was to implement a multidisciplinary approach combining the online monitoring of the fermentation process, the bioprocess management and two original and complementary approaches of statistical modeling (Box Behnken and GAM). This innovative strategy made it possible to (i) acquire a detailed understanding of the overall kinetics of volatile compound production and (ii) take into account both the direct and interaction effects of the parameters on the dynamics of aroma synthesis. This global view allows to go deeper in the understanding of the impact of the studied factors and to differentiate the metabolic and physicochemical effects.

Based on this original approach, the first objective of the present work was to investigate the impact of initial nitrogen, added nitrogen and temperature on the whole aroma production kinetics. The second objective was to “quantify” (weight and sign of variation) these effects on the volatile concentrations at the end of fermentation.

First, the analysis of the fermentation kinetics was studied and showed a significant impact of the three factors on its shape. All the results found are consistent with previous studies and confirm the robustness of the statistical analyses related to this study. For example, we confirm that higher initial and added nitrogen contents as well as increased temperature enabled to reduce fermentation time, as shown by Torija et al. (2003), Sablayrolles (2009), and Godillot et al. (2022).

In a second step, we focused on the results of the aromas online monitoring. The analysis of the production kinetics of propanol was more thorough because of its strong reactivity to the studied factors and of its atypical chronology of synthesis compared to the other aromas.

**FIGURE 6:**
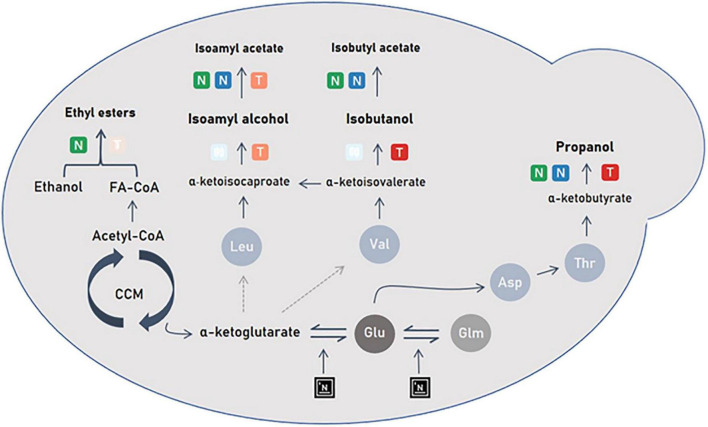
Scheme representing the impact of nitrogen and temperature management on yeast metabolism. NO is represented by a green gradient; Nadd by a blue gradient; Temp by a red gradient. The added nitrogen will act at the α-ketoglutarate node and induce a change in the flow of amino acid synthesis that affects aromas synthesis.

The parameter that had the highest impact on both propanol production kinetics and final content is added nitrogen (Figure 5 and Table 3). From a kinetic point of view, it was observed that the production of propanol restarts instantaneously after a nitrogen addition in stationary phase and with a higher yield than in growth phase (Figure 5). Indeed, as already noted in previous works (Seguinot et al., 2018; Godillot et al., 2022), an addition of nitrogen during the stationary phase is more “efficient” to increase propanol production compared to an initial addition. This specific relationship between propanol production and nitrogen addition can be explained by the following metabolic mechanisms: at first, the addition of nitrogen during fermentation will activate the genes responsible for amino acid synthesis (Godillot et al., 2022). Based on this stimulation of nitrogen metabolism, it is conceivable that the addition of ammonium during the stationary phase would directly activate the synthesis of aspartate *via* the glutamate node, notably because the most produced amino acids by central carbon metabolism are glutamate and aspartate (Crépin et al., 2017). Aspartate being the precursor of threonine, the concentration of this last amino acid would increase and finally result in a significantly increased synthesis of propanol (Figure 6). Possibly, the direct relation between propanol production and nitrogen consumption, in contrast to other higher alcohols, may be linked to a lower demand for threonine in proteins compared to other amino acids (such as leucine or valine for example) (Crépin et al., 2017). Propanol would thus act as a “safety valve,” preventing the accumulation of α-ketobutyrate and threonine in the intracellular medium (Mouret et al., 2014a).

In the present study, the increase in temperature coupled with an addition of nitrogen in stationary phase induced an increase in propanol production. Indeed, when the temperature is increased, so is cell activity (Sablayrolles, 2009). The genes responsible for the synthesis of α-ketoacids (*BAT1*) to higher alcohols (*THI3*, *ARO10*, *ADH2/3/5*) are overexpressed (Molina et al., 2007). This would explain that high fermentation temperatures promote the synthesis of higher alcohols (Figure 6). Conversely, no impact of temperature has been reported in the literature. In these works, nitrogen additions were done at the start of the process and not during the stationary phase. So, it could be thought that the temperature effect in the present study originated from a combined positive stimulation of synthesis of α-ketoacids (*BAT1*) to higher alcohols by both high temperature and nitrogen addition during the stationary phase.

To our knowledge, this is the first time that that propanol is shown sensitive to both nitrogen assimilation and temperature increase and to the interaction of these two factors. The effect of added nitrogen on propanol concentration is greater at higher temperatures than at lower temperatures, as is the effect on fermentation rate. The dynamics of the propanol production rate are very close to those of the CO_2_ release rate. Thus, propanol can be considered not only as a marker of nitrogen availability in the medium but also as a marker of cellular activity.

In contrast to propanol, isoamyl alcohol and isobutanol had a relatively different behavior with respect to the different factors studied in this study. The production kinetics of isoamyl alcohol and isobutanol are influenced by the initial nitrogen content: a high initial nitrogen concentration results in lower production for these two alcohols. Moreover, for isoamyl alcohol, we found a significant and negative quadratic effect of initial nitrogen showing an optimum at 140 mg/L of initial nitrogen (Figure 1).

Leucine and valine are amino acids (precursors of isoamyl alcohol and isobutanol, respectively), which in the growth phase, will be directly used for protein synthesis. Unlike threonine, the share of catabolism of these amino acids is low. Crépin et al. (2017) showed that the synthesis of isobutanol depends mainly on valine produced by CCM (Central Carbon Metabolism) and not on its assimilation in the medium. For isoamyl alcohol, synthesis depends mainly on the assimilation of leucine from the extracellular medium and its transformation into α-ketoisocaproate. During the exponential phase, the part of assimilated leucine and synthesized valine will go toward protein synthesis to meet cell demands. The flow toward the synthesis of higher alcohols is then weak compared to the demand in amino acids. A high concentration of initial nitrogen in the must implies a high demand for proteins, which reduces the synthesis of these two higher alcohols. Similar to the initial nitrogen content, added nitrogen also has a significant impact on the production of these two alcohols (Figure 1 and Table 3) and does not stimulate their production. As mentioned above, the demand for amino acids is once again stimulated and the flow goes toward the synthesis of proteins and not higher alcohols (Crépin et al., 2017).

The production kinetics of isoamyl alcohol and isobutanol are positively impacted by temperatures above or equal to 23°C. Moreover, the predictions of the Box-Behnken design are in line with the GAM modeling with a positive effect of temperature on their final productions. However, the two alcohols do not display the same behavior toward temperature. For isoamyl alcohol, an optimum appears at 23°C while the ideal temperature for isobutanol production is 28°C. This can be explained by a different regulation of the expression of the genes responsible for their synthesis and thus by a different management of the α-ketoisocaproate and α-ketoisovalerate pool between 18 and 28°C. The synthesis of these two higher alcohols therefore depends on CCM activation by temperature (Figure 6).

The production kinetics of isoamyl acetate, whether in liquid or in terms of total production, are impacted by the interaction of the three factors studied. These interactions are particularly important and mask some effects previously reported in the literature, such as the simple effect of initial nitrogen concentration (Hernandez-Orte et al., 2006; Rollero et al., 2015; Seguinot et al., 2018; Godillot et al., 2022). Indeed, depending on the concentration of added nitrogen or fermentation temperature, a high concentration of initial nitrogen is not necessarily associated to a very important isoamyl acetate production. The impact of one of the factors on the synthesis of this compound is therefore dependent on the value of the others. This clearly demonstrates that the management of the synthesis of this ester is multi-parametric. Moreover, such an observation shows that, even if it is important to study the effect of a single parameter on the fermentation process, it is also necessary to evaluate the possible interaction between different factors, as it is the case in the present work.

The aromatic signature of isoamyl acetate is characterized by a change in slope following the addition of nitrogen reflecting an acceleration of its production (Figure 3). As shown in previous studies, high concentrations of added nitrogen promote the synthesis of acetate esters (Seguinot et al., 2018; Godillot et al., 2022). Nitrogen addition during fermentation allows the activation of the *ATF2* gene responsible for the synthesis of acetate esters from higher alcohols (Seguinot et al., 2018; Godillot et al., 2022). This overexpression provokes an increase in the conversion of isoamyl alcohol into its ester and eventually a higher isoamyl acetate production (Figure 6).

Temperature has a negative impact on the production kinetics of isoamyl acetate on two aspects: physical and biological. Indeed, excess temperatures have a negative impact on the accumulation of this compound in the liquid phase since they will induce losses by evaporation (Figure 3E). Remarkably, this negative effect of temperature is also found for total production, indicating that isoamyl acetate synthesis by yeast is not optimal at 28°C (Figure 3E). All statistical analyses performed in the present work pointed out that, under our conditions, the ideal temperature for isoamyl acetate synthesis was 23°C (Figure 3D). An interesting observation is that it is also at this temperature that the synthesis of isoamyl alcohol is highest (Figure 2C). A possible explanation to this situation is that, at 23°C, yeast is in an ideal physiological state for the synthesis of higher alcohols and for the bioconversion of higher alcohols into esters, resulting therefore in a high acetate esters production (Figure 3F).

Concerning ethyl esters, we observed that their production kinetics were enhanced by high initial concentrations of nitrogen (Figure 4); nevertheless, the “intensity” of this effect is rather low as no significant effect of initial nitrogen on the final concentration of these esters could be evidenced (Table 3). Moreover, the synthesis of the two ethyl esters was not impacted by nitrogen addition during the stationary phase, as already shown in previous studies (Seguinot et al., 2018; Godillot et al., 2022). The lack of effects of added nitrogen on ethyl ester production is not surprising since their synthesis depends on fatty acids and thus on the lipid pathway (Barbosa et al., 2009; Seguinot et al., 2018).

This work clearly demonstrates that ethyl esters are highly impacted by temperature. We find a significant negative effect of temperature on production kinetics in the liquid. Indeed, as ethyl esters are particularly volatile and hydrophobic compounds, high temperatures induce a strong evaporation from the liquid to the gas for temperatures above 18°C. These results are in agreement with many previous studies (Morakul et al., 2013; Mouret et al., 2014a; Rollero et al., 2015). Most strikingly, the same negative effect is found for the total production of ethyl hexanoate and ethyl octanoate, indicating that effect of temperature on the production of ethyl esters is both physical and biological. Their synthesis by yeast would only be optimal at temperatures ranging from 18 to 23°C. Consequently, the fact that the highest accumulation of these volatile compounds was obtained at 18°C results from two phenomena: 1/a low evaporation and 2/a high yeast production. Concerning the biological production of these molecules, a previous study showed that the genes responsible for the activation of fatty acids by acetyl-CoA (*MCT1*, *ETR1*, *OAR1*) are overexpressed at low temperature (Beltran et al., 2006), in which case the quantity of available acyl-CoA precursors would be higher, resulting in an increased synthesis of the corresponding ethyl esters (Figure 6).

## 5. Conclusion

The objective of this study was to understand the simultaneous impact of three factors on fermentation kinetics and aroma synthesis kinetics during fermentation. To do so, we combined precise determination of production dynamics, mass balances and the use of two complementary statistical analysis methods: GAM and Box-Behnken.

This innovative approach allowed us to study the weight of each of the factors on the shape of the kinetics on the one hand, and on specific kinetics parameters (difference between Vmax; final concentration in the liquid), on the other hand. It was found that the production of the different families of aromas through yeast metabolism was regulated differently. But this global and combined approach also allowed identifying original results of primary metabolic interest: (i) propanol can be considered as a marker of cellular activity; (ii) temperature has both biological and physical impacts on esters production dynamics and (iii) temperature leading to the maximal liquid accumulation was different for the two classes of esters.

In future works, it would be interesting, using this combination of analyses, to study the dual effect of temperature in greater depth and to integrate a transcriptomic approach to further understand the related biological phenomena.

## Data availability statement

The datasets presented in this study can be found in online repositories. The names of the repository/repositories and accession number(s) can be found below: https://doi.org/10.57745/MUFRM9.

## Author contributions

MP and EA designed the experimental strategy to perform all fermentations of this study and developed the associated tools including automatic nitrogen addition. YS developed the analytical method and calibration procedure for the online GC equipment for volatile compound monitoring. IS and MB designed the Box-Behnken experimental design, built the two GAM models, and performed the associated statistical analysis. JG and CB implemented all the experiments. JG drafted the manuscript. J-RM, VF and JG performed the interpretation of all the data conceived and designed the overall study. J-RM, VF, and J-MS revised the manuscript. All authors read and approved the final version of the manuscript.
